# Gut microbiota and serum metabolomics in children with human parainfluenza virus infection

**DOI:** 10.3389/fcimb.2026.1941674

**Published:** 2026-10-05

**Authors:** Xinghan Ren, Zhuoying Liu, Liuxuan Huang, Xiaotong Lai, Binyu Wu, Tianyu Bai, Xinyue Shen, Weijian Liang, Zhongting Dai, Huanqiong Li, Rong Zhou, Tiantian Liu

**Affiliations:** 1 School of Public Health, Guangdong Pharmaceutical University, Guangzhou, Guangdong, China; 2 Foshan Sanshui District People’s Hospital (Sanshui Hospital, Zhujiang Hospital, Southern Medical University), Foshan, Guangdong, China

**Keywords:** children, gut microbiota, human parainfluenza virus, metabolomics, multi-omics association, pathogenic mechanisms

## Abstract

**Objective:**

Based on gut microbiota and serum untargeted metabolomics analyses, this study investigated changes in gut microbiota structure and serum metabolic profiles in children infected with human parainfluenza virus (HPIV). By integrating multi-omics data, we aimed to explore interactions between gut dysbiosis and serum metabolic disorders, and their potential roles in HPIV pathogenesis, to provide preliminary evidence for microbiota--targeted and metabolic intervention strategies.

**Methods:**

Clinical data and laboratory results were collected from 230 hospitalized children (<14 years) with HPIV infection. Subsequently, 31 HPIV-infected children and 12 healthy controls were enrolled. Fecal samples were analyzed via 16S rRNA gene sequencing, and serum samples via liquid chromatography-tandem mass spectrometry (LC-MS/MS)-based untargeted metabolomics, followed by integrated multi-omics analysis.

**Results:**

Children with severe HPIV infection had longer hospital stays and higher frequencies of wheezing, tachypnea, dyspnea, and vomiting. HPIV infection was associated with gut dysbiosis: opportunistic pathogens (*Anaerococcus*, *Peptoniphilus*, *Finegoldia*) were enriched, while beneficial commensals (*Blautia*, *Bifidobacterium*, *Faecalibacterium*) were reduced. Metabolomics identified 579 upregulated and 211 downregulated metabolites, primarily enriched in alpha-linolenic acid, butanoate, glycerophospholipid, starch/sucrose, and glycine/serine/threonine metabolism pathways. Correlation analysis showed that enriched opportunistic pathogens positively correlated with pro-inflammatory lipids (e.g., acetoacetic acid, 3-hydroxybutyric acid) and negatively with protective metabolites (e.g., alpha-linolenic acid, sucrose, L-threonine), while *Blautia* showed opposite patterns. The four key genera and five metabolites demonstrated exploratory discriminatory capacity for HPIV infection (AUC: 0.81 and 0.98, respectively), serving as candidate biomarkers for further validation.

**Conclusion:**

HPIV infection may be associated with gut microbiota dysbiosis and serum metabolic disturbances in children, potentially via the gut–lung axis, and these alterations may be involved in modulating pulmonary antiviral immune and inflammatory responses. This multi-omics study provides preliminary evidence for future research on prevention and treatment strategies targeting the gut microbiota or metabolic pathways in children with HPIV infection.

## 1 Introduction

Human parainfluenza virus (HPIV) is a major cause of respiratory disease-related hospitalizations among infants and young children and a significant pathogen responsible for acute respiratory infections in young children. In the United States, it is estimated that approximately 30,000 children are hospitalized annually due to HPIV infection (Oh et al., 2021). HPIV is primarily transmitted through respiratory secretions. Since the human body does not develop long-lasting immunity after infection, repeated infections are common, though symptoms are relatively mild upon reinfection. Due to their young age and weaker immune systems, children experience rapid disease progression and are more prone to severe infections. Severe acute respiratory infections are a leading cause of hospitalization and death among children worldwide. As the second most common pathogen causing respiratory infections, HPIV has a detection rate second only to RSV. Reports indicate that HPIV infections account for up to 4% of deaths from acute lower respiratory tract infections; it is estimated that approximately 35,000 children worldwide died from this cause in 2018 (Wang et al., 2021).

In recent years, the ‘gut–lung axis’ theory has provided a new perspective for understanding the pathogenesis of respiratory viral infections. The gut microbiota promotes mucosal development, aids in nutrient metabolism, and defends against pathogenic bacteria, playing a key role in maintaining the intestinal environment. Groves et al (Groves et al., 2020). found that the composition of the gut microbiota changes during respiratory syncytial virus (RSV) and influenza A virus infections, accompanied by common symptoms such as weight loss and loss of appetite. In some cases, the binding of viruses to bacterial surfaces can facilitate viral infection. For example, lipopolysaccharide (LPS) can bind to viruses such as poliovirus and mouse mammary tumor virus (MMTV), thereby enhancing the viruses’ ability to attach to cells, maintaining their infectivity at high temperatures, and increasing their capacity for immune evasion and transmission (Robinson et al., 2014). More importantly, the role of the microbiota extends beyond the gastrointestinal tract to influence other systems by modulating immune responses. For example, both RSV and SARS-CoV-2 infections significantly alter patients’ fecal microbiomes, resulting in a reduction in the proportion of gut microbiota with immunomodulatory potential (Groves et al., 2018; Zuo et al., 2020). Both local (respiratory tract) and distal (gut) microbiota influence the pulmonary immune response to influenza A virus (IAV) infection. Yitbarek et al (Yitbarek et al., 2018). demonstrated that the gut microbiota may initiate an immune response against the avian influenza virus (AIV) H9N2 subtype via a type I interferon-dependent mechanism.

Gut dysbiosis may also affect host metabolism and adaptive immunity. Short-chain fatty acids (SCFAs) are bacterial metabolites produced by specific gut microbes through the metabolism of soluble fiber. Studies have shown that they improve gut homeostasis by activating G protein-coupled receptors (GPCRs) such as GPR41, GPR43, or GPR109a (Vieira et al., 2017) and by inhibiting histone deacetylases (Corrêa-Oliveira et al., 2016). Recent studies have revealed that the composition of short-chain fatty acids is closely associated with antiviral immunity against certain viruses. For example, the intake of dietary fiber (the raw material for synthesizing short-chain fatty acids in pregnant women) can protect newborns from severe RSV infections (Ferolla et al., 2013); in animal studies, a high-fiber diet has also been observed to provide protection against rotavirus infection (Yang et al., 2021). Antunes et al (Antunes et al., 2019). demonstrated that acetic acid is a key protective metabolite in rotavirus infection; it helps reduce viral load and pulmonary inflammation through a unique mechanism: by activating G protein-coupled receptor 43 (GPR43), acetic acid promotes the type I interferon response and the expression of interferon-stimulated genes in pulmonary epithelial cells. Certain metabolites may lead to adverse complications in patients with viral infections. Sinha et al (Sinha et al., 2019). demonstrated that gut dysbiosis caused by Human Immunodeficiency Virus (HIV) infection significantly elevates levels of carnitine-related metabolites and is closely associated with adverse cardiovascular events in these patients.

However, systematic studies on the ‘gut–lung axis’ and gut microbiota-mediated regulation of host metabolism in HPIV infection are currently lacking, with evidence in pediatric populations being particularly scarce. Based on existing research progress, this study enrolled hospitalized children with HPIV infection and conducted a combined analysis integrating gut microbiota profiling with serum untargeted metabolomics: systematically characterizing the gut microbiota composition in children with HPIV infection, quantitatively analyzing changes in serum metabolomic profiles, further exploring the associations between gut microbiota and serum metabolites, elucidating the core regulatory pathways in which both are involved, and screening for key biomarkers that can be used for disease diagnosis. This study provides an important theoretical basis for elucidating the molecular mechanisms by which the gut microbiota regulates lung injury and immune dysregulation in children with respiratory viral infections, while also offering new insights and a reference framework for research on the interactions between the microbiota and metabolism in respiratory viral infections.

## 2 Methods

### 2.1 Study participants

A total of 230 children under the age of 14 who were hospitalized with HPIV infection were recruited between January and May 2025. Throat swab or sputum samples were collected within 24–48 hours of admission, and HPIV infection was confirmed using multiplex polymerase chain reaction (PCR) combined with capillary electrophoresis fragment analysis. Fecal and serum samples were collected from 31 children with HPIV infection at admission for subsequent multi-omics analysis. Additionally, fecal and serum samples were collected from 12 healthy children (HCs) matched for age, sex, and BMI during routine physical examinations (these healthy control samples were also used in parallel studies conducted by our research team).

### 2.2 Inclusion and exclusion criteria for study subjects

For the disease group, eligible participants were hospitalized children aged 14 years or younger with clinically diagnosed and laboratory-confirmed HPIV infection. Written informed consent was obtained from guardians after full understanding of study aims, procedures and sample usage. Fecal and fasting venous blood samples were collected within 24 to 48 hours upon admission. Nasopharyngeal swabs were tested using a commercial 18-pathogen nucleic acid panel covering common respiratory pathogens, including 10 viruses, 3 atypical pathogens, and 4 bacteria. Patients were excluded if they received systemic antibiotics, antifungals, probiotics or prebiotics within four weeks before enrollment; had chronic diseases affecting gut microbiota or systemic metabolism; had congenital or acquired immunodeficiency or received immunosuppressive therapy; or presented with concurrent infections caused by other pathogens at enrollment.

Healthy children matched to the HPIV group by age, sex, and BMI were enrolled as controls. Controls had no recent respiratory or gastrointestinal infectious symptoms and normal physical examination results, with signed informed consent provided by guardians. Control children were excluded if they used antibiotics, antifungals, probiotics or prebiotics in the preceding four weeks, or had immune dysfunction, chronic underlying diseases or congenital developmental abnormalities.

### 2.3 Data collection and sample processing

Demographic information, clinical data at admission, and laboratory test results were collected from hospitalized children with HPIV infection. According to the Guidelines for the Management of Community-Acquired Pneumonia in Children (2024 Revision) (Subspecialty Group of Respiratory, the Society of Pediatrics, Chinese Medical Association et al., 2024), pneumonia severity was classified into mild and severe groups. Age was categorized into four groups: infancy (<1 year), early childhood (1–3 years), preschool (3–5 years), and school-age (≥5 years); place of residence was classified as urban or rural.

Fecal samples were collected from fresh stool using sterile collection tubes. After collection, the samples were immediately flash-frozen in liquid nitrogen for 2–3 minutes and stored at −80 °C until analysis.

Serum samples were collected by drawing 3 mL of venous blood in the morning on an empty stomach into EDTA-anticoagulated tubes. After clotting for 30 minutes at room temperature, the samples were centrifuged at 3000 rpm for 10 minutes. The supernatant was then aspirated and aliquoted into 1.5 mL sterile microcentrifuge tubes (200 μL per tube). The aliquoted samples were immediately flash-frozen in liquid nitrogen for 2–3 minutes and stored at −80 °C until analysis.

### 2.4 16S rRNA gene sequencing

Total genomic DNA was extracted from fecal samples using the FastPure Stool DNA Isolation Kit according to the manufacturer’s instructions. DNA integrity was assessed by 1% agarose gel electrophoresis, and DNA concentration and purity were measured using a NanoDrop 2000 spectrophotometer. Only samples with an A260/A280 ratio between 1.8 and 2.0 were used for subsequent library construction. The V3–V4 hypervariable region of the 16S rRNA gene was amplified by PCR with barcoded primers 338F (5′-ACTCCTACGGGAGGCAGCAG-3′) and 806R (5′-GGACTACHVGGGTWTCTAAT-3′). PCR was performed in a 20 μL reaction mixture, with thermal cycling conditions: initial denaturation at 95 °C for 3 min; 27 cycles of 95 °C for 30 s, 55 °C for 30 s, and 72 °C for 30 s; and a final extension at 72 °C for 10 min. PCR products were pooled, purified, and quantified. Purified amplicons were used to construct paired-end libraries with the NEXTFLEX^®^ Rapid DNA-Seq Kit. High-throughput sequencing was performed on the Illumina NextSeq 2000 PE300 platform.

Bioinformatic analysis was performed within the QIIME2 framework. Raw sequences were quality-filtered, merged, and denoised using the DADA2 plugin with default parameters to generate amplicon sequence variants (ASVs). ASVs annotated as chloroplast or mitochondrial sequences were removed. All samples were rarefied to 20,000 reads; after rarefaction, the average Good’s coverage reached 99.09%. Taxonomic assignment of ASVs was performed using the QIIME2 naive Bayes classifier against the SILVA 16S rRNA database (v138.2). Functional prediction was carried out using PICRUSt2 (v2.2.0) (Douglas et al., 2020). Negative blank controls (extraction blanks and PCR blanks) were processed alongside experimental samples throughout DNA extraction, PCR amplification and library construction to monitor reagent and environmental contamination. Negative controls yielded negligible sequencing reads and failed to generate sufficient DNA for library construction.

### 2.5 LC-MS/MS analysis

Equal volumes of metabolites from all samples were mixed to prepare quality control (QC) samples. A QC sample was inserted after every 5–10 sample runs to continuously monitor instrumental stability, peak reproducibility, and batch consistency. The instrument platform used for LC-MS/MS analysis was a Thermo Fisher UHPLC–Orbitrap Exploris 240 system (Han et al., 2024). Chromatographic separation was performed on an HSS T3 column (100 mm × 2.1 mm i.d., 1.8 µm) at 40 °C, with a flow rate of 0.40 mL/min. Mobile phase A consisted of water/acetonitrile (95:5, v/v) with 0.1% formic acid, and mobile phase B consisted of acetonitrile/isopropanol/water (47.5:47.5:5, v/v/v) with 0.1% formic acid. Mass spectrometry was performed in positive and negative ion scan modes over a mass range of m/z 70–1050. Ion spray voltages were set to 3500 V (positive) and −3000 V (negative); sheath gas and auxiliary heating gas were 50 arb and 13 arb, respectively; ion source temperature was 450 °C; and collision energy was cycled through 20, 40, and 60 V.

Raw metabolomic data were processed using Progenesis QI v3.0 for baseline filtering, peak picking, peak integration, retention time correction, and peak alignment, generating a data matrix containing retention time, mass-to-charge ratio (m/z), and peak intensity. Metabolite annotation was performed by matching experimental MS^1^ and MS/MS spectra against public databases (HMDB, Metlin) and an in-house database, with a precursor mass tolerance of < 10 ppm. Metabolite identification was assigned at MSI Level 2 (putatively annotated compounds) based on MS/MS spectral matching scores. To ensure analytical reliability, pooled QC samples and blank controls (extraction blanks without biological matrix) were included throughout each analytical batch. Multiple internal standards were applied to evaluate system performance; an RSD ≤ 20% for internal standard signals across all QC samples indicated stable instrument status.

For data preprocessing, missing values were imputed using multiple strategies, and normalization was performed using the sum normalization method. Metabolite features with RSD > 30% in QC samples were excluded to remove unstable variables, and log transformation was applied to improve data normality.

### 2.6 Multi-omics association analysis

Differential microbial features identified via 16S rRNA gene sequencing and differential serum metabolites screened by untargeted metabolomics were integrated. Spearman correlation analysis was conducted to assess associations between differential gut microbes and differential serum metabolites. Benjamini–Hochberg FDR correction was applied to adjust *P*-values for multiple comparisons, and only correlations with FDR *q*-value < 0.05 were retained for interpretation and visualization.

### 2.7 Data processing and statistical analysis

Clinical characteristics of hospitalized children with HPIV infection were analyzed using Stata 18.0. The normality of continuous variables was assessed using the Shapiro–Wilk test. As all continuous variables deviated significantly from a normal distribution (*P* < 0.05), they were summarized as medians with interquartile ranges (IQR) and compared between groups using the Mann–Whitney U test. Categorical variables were summarized as frequencies with percentages and compared using the Pearson chi-square test, or Fisher’s exact test when any expected cell count was < 5. All reported *P*-values are nominal and were not adjusted for multiple comparisons. A two-sided *P* < 0.05 was considered statistically significant.

For data obtained from 16S rRNA gene sequencing, alpha diversity indices (Chao1 and Shannon) were calculated using Mothur, with differences between groups assessed by the Wilcoxon rank-sum test. Differential microbial taxa between groups were identified using the Wilcoxon rank-sum test. To account for multiple comparisons, the Benjamini-Hochberg false discovery rate (FDR) correction was applied, and taxa with an FDR *q*-value < 0.05 were considered significantly different. Beta diversity was assessed using principal coordinate analysis (PCoA) based on Bray–Curtis distances and principal component analysis (PCA), and statistical significance was evaluated by PERMANOVA (999 permutations), with sex and age included as covariates. Homogeneity of multivariate dispersions was confirmed using the betadisper test (*P* > 0.05) prior to PERMANOVA. Linear Discriminant Analysis Effect Size (LEfSe) analysis (LDA scores > 2, *P* < 0.05) was used to identify bacterial taxa with significant differences in genus-level abundance between groups. Genera with Spearman correlation |*r*| ≥ 0.6 and *P* < 0.05 were selected to construct a correlation network diagram. Software and versions applied for 16S rRNA gene sequencing data analysis are listed in **Supplementary Table 1**.

For data obtained via LC-MS/MS, the ropls package (v1.6.2) in R was used for principal component analysis (PCA) and orthogonal partial least squares discriminant analysis (OPLS-DA) on the preprocessed data matrix, with model stability assessed by 7-fold cross-validation. Significantly differential metabolites were identified based on variable importance in the projection (VIP values > 1) obtained from the OPLS-DA model and FDR *q*-value < 0.05. Pathway annotation and enrichment analysis were performed using the KEGG database and Fisher’s exact test (scipy.stats, Python), respectively, with FDR correction for multiple comparisons. Pathways with FDR *q*-value < 0.05 were considered significantly enriched. Software versions are listed in **Supplementary Table 2**.

To exploratorily assess whether hospitalization-related factors contributed to the observed gut microbial and metabolic alterations, Spearman’s rank correlation coefficients were calculated between the core differential genera/metabolites and clinical proxies of hospitalization stress (length of hospital stay and pneumonia severity) within the HPIV group. To estimate the precision of the correlation coefficients, 95% confidence intervals were calculated using bootstrap resampling with 1,000 replications (percentile method).

## 3 Results

### 3.1 Clinical characteristics of hospitalized children with HPIV infection

The baseline clinical and laboratory characteristics of the study population are summarized in **Table 1**. Compared with patients with mild illness, those with severe illness were younger, had longer hospital stays, and were more likely to experience vomiting, wheezing, tachypnea, and dyspnea (all *P* < 0.05). Laboratory parameters also differed significantly by disease severity: PLT and WBC counts were significantly elevated in the severe group, whereas HGB levels were significantly lower (all *P* < 0.05). In the multivariable logistic regression model adjusting for sex, age, and residence, dyspnea remained the strongest independent risk factor for severe disease (aOR = 14.56, 95% CI: 6.19–34.23, *P* < 0.001), followed by vomiting (aOR = 2.58, 95% CI: 1.08–6.14, *P* = 0.033) and lower HGB level (aOR = 0.98 per 1 g/L increase, 95% CI: 0.95–1.00, *P* = 0.045) (**Supplementary Table 3**).

**Table 1:** Clinical characteristics of 230 hospitalized children with HPIV infection.

Variable	Mild (n = 164)	Severe (n = 66)	*P*-value
Sex, n (%)			0.645
Male	99 (60.37)	42 (63.64)	
Female	65 (39.63)	24 (36.36)	
Place of residence, n (%)			0.190
Urban	94 (57.32)	44 (66.67)	
Rural	70 (42.68)	22 (33.33)	
Age (years), n (%)			0.004
<1 year	34 (20.73)	26 (39.39)	
1 to < 3 years	53 (32.32)	24 (36.36)	
3 to < 5 years	52 (31.71)	13 (19.70)	
≥ 5 years	25 (15.24)	3 (4.55)	
Length of hospitalization (days), median (IQR)	4 (4, 6)	5 (4, 7)	0.001
Cough duration (days), median (IQR)	5 (3, 10)	5 (2, 7)	0.571
Fever duration (days), median (IQR)	3 (1, 4)	3 (1, 5)	0.634
Clinical symptoms, n (%)			
Sputum production	111 (67.68)	49 (74.27)	0.328
Nasal congestion	107 (65.24)	51 (77.27)	0.075
Rhinorrhea	109 (66.46)	47 (71.21)	0.486
Convulsion	8 (4.88)	5 (7.58)	0.528
Vomiting	21 (12.80)	17 (25.76)	0.017
Wheezing	17 (10.37)	18 (27.27)	0.001
Tachypnea	5 (3.05)	20 (30.30)	<0.001
Dyspnea	14 (8.54)	37 (56.06)	<0.001
Laboratory indicators, median (IQR)			
HGB (g/L)	120 (113, 127)	113 (103, 119)	<0.001
PLT (10^9^/L)	270 (218, 362)	323 (250, 437)	0.005
WBC (10^9^/L)	8.57 (6.10, 11.26)	10.68 (7.12, 13.99)	0.005
NEUT (10^9^/L)	3.58 (2.25, 5.78)	4.23 (2.79, 6.54)	0.105
LYMPH (10^9^/L)	3.51 (2.12, 4.96)	4.16 (2.47, 6.29)	0.058
CRP (mg/L)	4.60 (1.15, 11.51)	6.32 (0.65, 16.95)	0.289
PCT (ng/mL)	0.13 (0.07, 0.25)	0.13 (0.08, 0.34)	0.401
IL-6 (pg/mL)	12.30 (5.64, 22.50)	19.44 (5.86, 36.80)	0.097

HGB, hemoglobin; PLT, platelets; WBC, white blood cell; NEUT, neutrophil; LYMPH, lymphocyte; CRP, C-reactive protein; PCT, procalcitonin; IL-6, interleukin-6. *P* < 0.05 was considered statistically significant for comparisons between mild and severe groups.

A total of 31 hospitalized children with HPIV infection were enrolled in this study for subsequent 16S rRNA gene sequencing and LC-MS/MS metabolomic profiling. To assess whether these 31 cases were representative of the overall HPIV cohort, we compared their clinical characteristics with those of the remaining 199 non-included patients (**Supplementary Table 4**). No statistically significant differences were observed between the two groups across all demographic, clinical, and laboratory variables (all *P* > 0.05), indicating that the included sample is reasonably representative of the overall HPIV cohort.

### 3.2 Gut microbiota dysbiosis in children with HPIV infection

In this study, we analyzed the biological composition of the gut microbiota in hospitalized children with HPIV infection by performing 16S rRNA gene sequencing on stool samples. After merging and filtering, a total of 2,400,626 effective reads were obtained (**Supplementary Table 5**). Rarefaction curves for community richness (Sobs index) and diversity (Shannon index) approached a plateau, indicating that the sequencing depth adequately covered most microbial diversity across all samples (**Supplementary Figure 1**).

The core species accumulation curves showed a gradual flattening trend in both the HPIV and HCs groups, indicating that the sample size was sufficient to characterize the core microbial communities within each group (**Figure 1A**). Alpha diversity, as measured by the Shannon index (diversity) and Chao1 index (richness), did not differ significantly between the HPIV and HCs groups (**Figures 1B, C**). The number of ASVs in the HPIV group and the HCs group was 223 and 157, respectively, with 123 shared ASVs between the two groups (**Figure 1D**). PCA and PCoA (based on Bray–Curtis distances) revealed distinct separation in fecal microbial community composition between the HPIV and HCs groups (**Figures 1E, F**). PERMANOVA confirmed significant differences in community structure between the two groups (*F* = 17.77, *R*² = 0.296, *P* = 0.001; **Supplementary Table 6**), while sex (*P* = 0.614) and age (*P* = 0.099) were not significant covariates. Hierarchical cluster analysis at the genus level revealed distinct clustering between the HPIV and HCs groups, suggesting that HPIV infection was associated with alterations in the structure of the dominant gut microbiota (**Figure 1G**). Results from the gut microbiota health index (GMHI) and microbiota dysbiosis index (MDI) showed that HPIV infection was associated with a decline in fecal microbial health and severe dysbiosis (**Figures 1H, I**).

**Figure 1:**
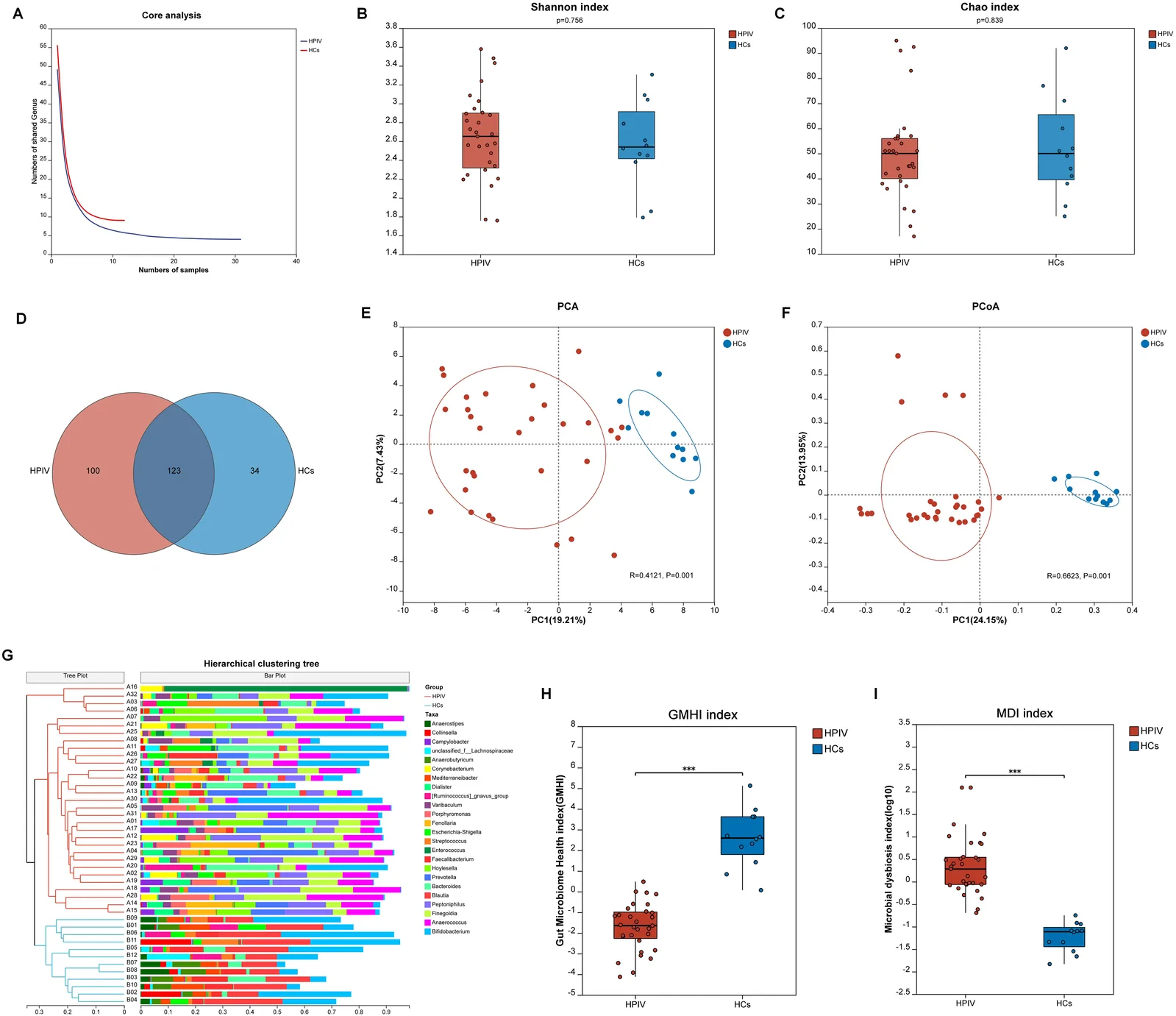
Changes in fecal microbial diversity and community structure between the HPIV and HCs groups. **(A)** Core species accumulation curve confirming adequate sample size. **(B, C)** Comparisons of Chao1 and Shannon α-diversity indices. **(D)** Venn diagram illustrating shared and unique ASVs. **(E, F)** PCA and PCoA ordination plots based on the Bray–Curtis distance for β-diversity. **(G)** Hierarchical clustering tree at the genus level. **(H, I)** Comparisons of GMHI and MDI dysbiosis indices. Statistical significance was set at *P* < 0.05; *P < 0.05, **P < 0.01, ***P < 0.001.

### 3.3 Differences in dominant gut bacterial genera among children with HPIV infection

To investigate changes in the fecal microbiota of hospitalized children with HPIV infection, we evaluated the relative abundances of bacterial genera between the HPIV and healthy control groups.

The community heatmap illustrated the distribution and trends of fecal microbiota in the HPIV group and the HCs group; HPIV infection was associated with alterations in microbial composition, resulting in significant changes in dominant bacterial genera (**Figure 2A**). LEfSe analysis revealed that the gut microbiota of HPIV patients was dominated by *Anaerococcus*, *Finegoldia*, *Peptoniphilus*, and *Prevotella*, whereas that of the HCs group was dominated by *Bifidobacterium*, *Blautia*, *Faecalibacterium*, *Anaerobutyricum*, and *Mediterraneibacter* (**Figure 2B**). Regarding specific differential microbial communities, compared with healthy children, the relative abundances of genera such as *Bifidobacterium*, *Blautia*, *Faecalibacterium*, *Streptococcus*, *Mediterraneibacter*, *Anaerobutyricum*, and *Collinsella* were significantly reduced in children infected with HPIV, while the relative abundances of genera such as *Anaerococcus*, *Finegoldia*, *Peptoniphilus*, *Prevotella*, and *Hoylesella* were significantly elevated (**Figures 2C, D**).

**Figure 2:**
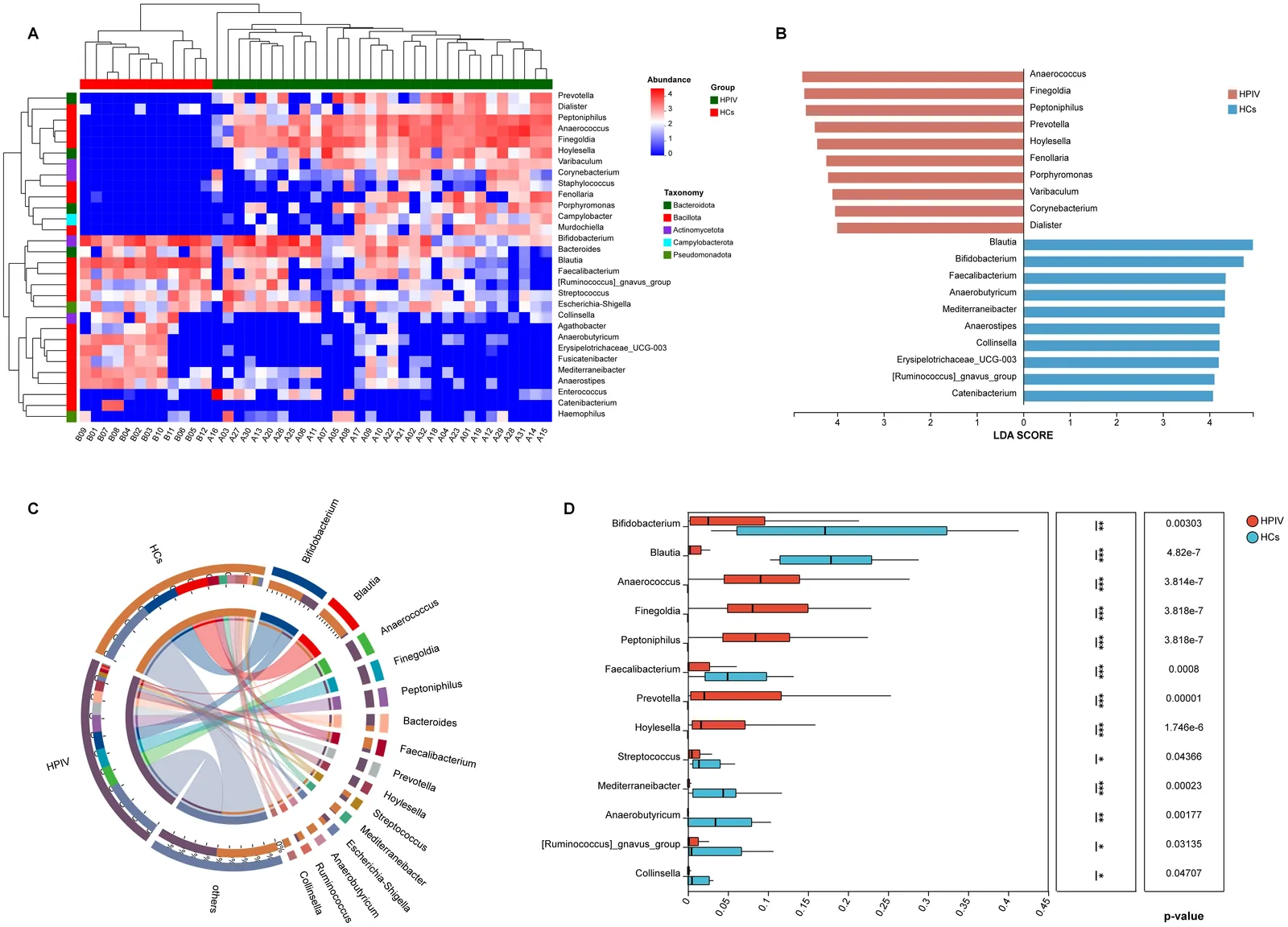
Abundance distribution of dominant fecal microbial genera between the HPIV and HCs groups. **(A)** Community abundance heatmap of bacterial genera. **(B)** LEfSe analysis screening for differential bacterial genera at the genus level (LDA score > 3.5). **(C, D)** Circos diagrams illustrating the distribution of differential bacterial genera. Statistical significance was set at *P* < 0.05; *P < 0.05, **P < 0.01, ***P < 0.001.

### 3.4 HPIV infection alters metabolite profiles in pediatric patients

After preprocessing the raw metabolomics data, a total of 2,530 metabolites were identified, including 1,124 in positive ion mode (LC-MS/MS (+)) and 1,406 in negative ion mode (LC-MS/MS (-)).

To assess the impact of HPIV infection on the serum metabolome, we analyzed metabolites in serum samples from the HPIV group and the HCs group. A Partial Least Squares Discriminant Analysis (PLS-DA) model was employed, with cross-validation to assess model robustness and evaluate potential overfitting. The PLS-DA score plots demonstrated that metabolite profiles could clearly distinguish the HPIV group from the HCs group (**Figures 3A, D**). Cross-validation results indicated that the model exhibited robust predictive performance (**Figures 3B, E**). PCA revealed significantly different distributions between the two groups in both positive and negative ion modes (**Figures 3C, F**). The Venn diagram showed that the HPIV group had 319 unique metabolites, while the HCs group had 71 unique metabolites, with 2,463 metabolites shared between the two groups (**Figure 3G**). The clustering heatmap revealed distinct clustering separation in serum metabolite composition between the two groups, suggesting that HPIV infection was associated with alterations in the serum metabolite profile in pediatric patients (**Figure 3H**).

**Figure 3:**
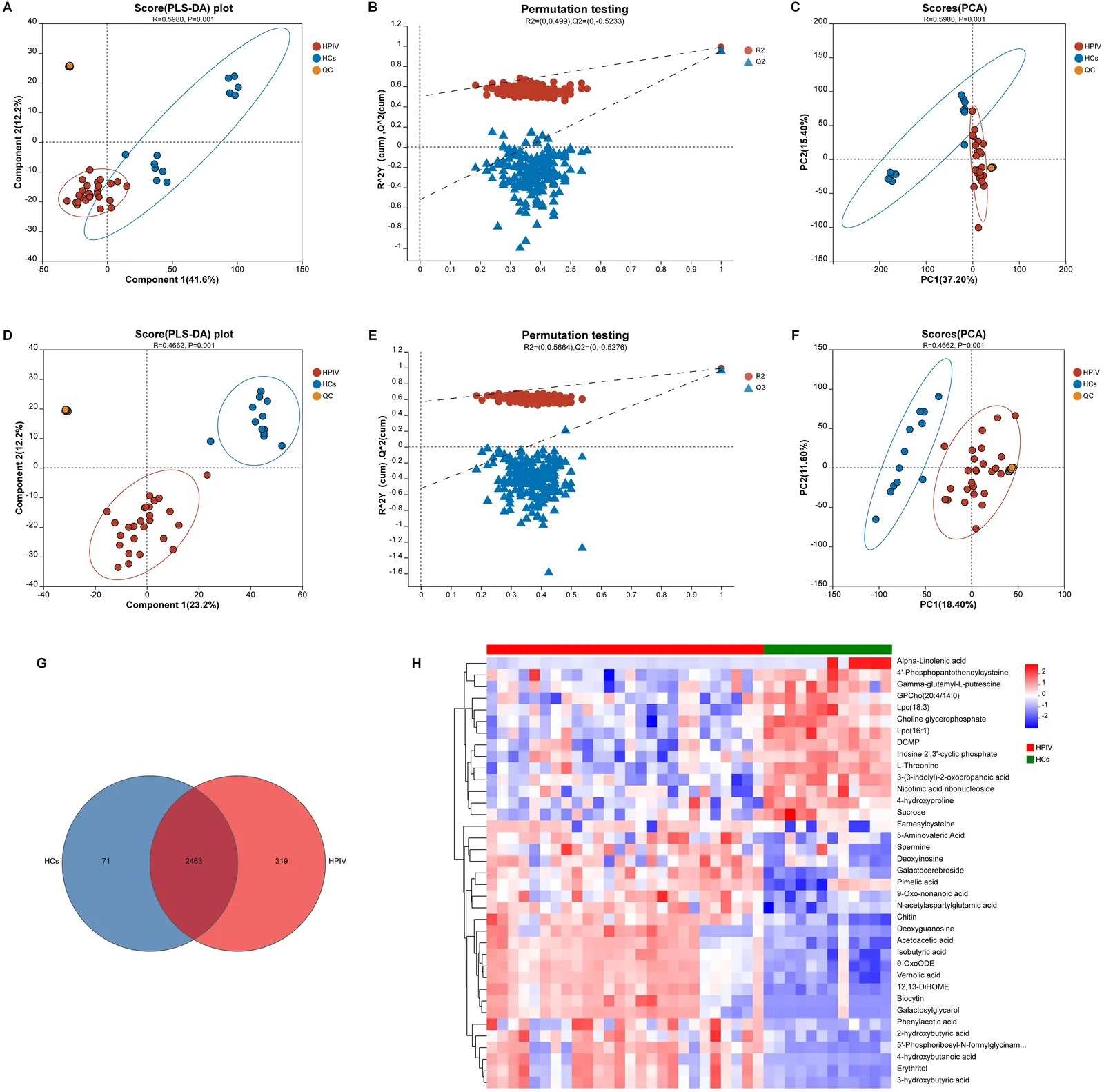
Distribution characteristics of serum metabolites between the HPIV and HCs groups. **(A–C)** PLS-DA score plot, seven-fold cross-validation result, and PCA scatter plot in positive ion mode. **(D–F)** PLS-DA score plot, 200-fold bootstrap test result, and PCA scatter plot in negative ion mode. **(G)** Venn diagram of shared and unique serum metabolites. **(H)** Clustering heatmap of serum metabolite profiles. Statistical significance was set at *P* < 0.05.

### 3.5 Analysis of metabolite differences in the serum of children with HPIV infection

VIP values determined by the OPLS-DA model reflected the individual contribution of each metabolite’s abundance to the observed intergroup differences. Variables with VIP values > 1 and FDR *q*-value < 0.05 were identified as differential metabolites. A total of 37 key serum metabolites were ultimately identified, and their VIP values and diagnostic performance were evaluated (**Supplementary Tables 7**, **8**).

The OPLS-DA score plot showed that the metabolite profiles of HPIV patients exhibited significant differences from those of healthy controls, with the model suggesting adequate fit and predictive performance (**Figures 4A, B**). The volcano plot visualized that 579 metabolites were significantly upregulated and 211 metabolites were significantly downregulated in the HPIV group (**Figure 4C**). The VIP bubble plot illustrated the influence of each metabolite’s expression pattern on sample classification based on VIP values derived from the first principal component (**Figure 4D**). Compared with the HCs group, serum metabolites such as galactosylglycerol, biocytin, 4-hydroxybutanoic acid, and phenylacetic acid were significantly upregulated in the HPIV group, while metabolites such as 4′-phosphopantothenoylcysteine, nicotinic acid ribonucleoside, alpha-linolenic acid, and inosine 2′,3′-cyclic phosphate were significantly downregulated. The relationships among the differential metabolites were evaluated using Spearman correlation analysis (**Figure 4E**). Significant positive correlations were observed among metabolites enriched in the HPIV group, whereas negative correlations were observed between the differential metabolites in the HPIV group and those in the HCs group.

**Figure 4:**
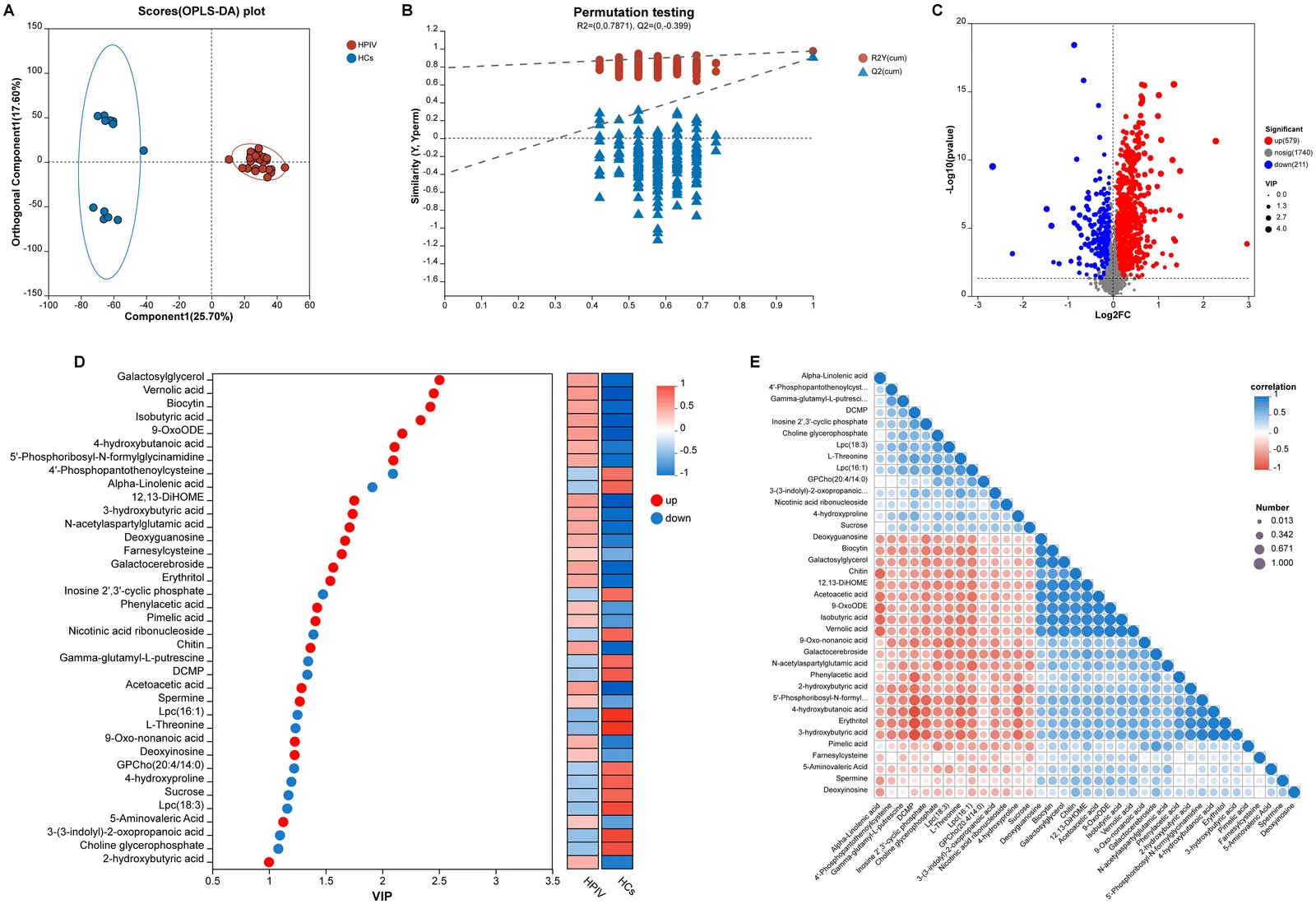
Identification and screening of serum differential metabolites between the HPIV and HCs groups. **(A, B)** OPLS-DA score plot and corresponding seven-fold cross-validation result. **(C)** Volcano plot of serum metabolites. **(D)** VIP bubble plot illustrating the importance of differential metabolites. **(E)** Spearman correlation heatmap of differential metabolites. Statistical significance was set at *P* < 0.05.

### 3.6 Enrichment analysis of serum metabolites in children with HPIV infection

Based on the differential metabolites between the HPIV and HCs groups, KEGG pathway enrichment and topological analyses were performed on metabolic pathways. The most relevant pathways were selected based on statistical significance (*P* < 0.05) and biological relevance (impact values > 0.1) (**Figures 5A, B**). Compared with the HCs group, the differential serum metabolites in the HPIV group were primarily enriched in pathways related to alpha-linolenic acid metabolism, butanoate metabolism, glycerophospholipid metabolism, starch and sucrose metabolism, and glycine, serine, and threonine metabolism.

**Figure 5:**
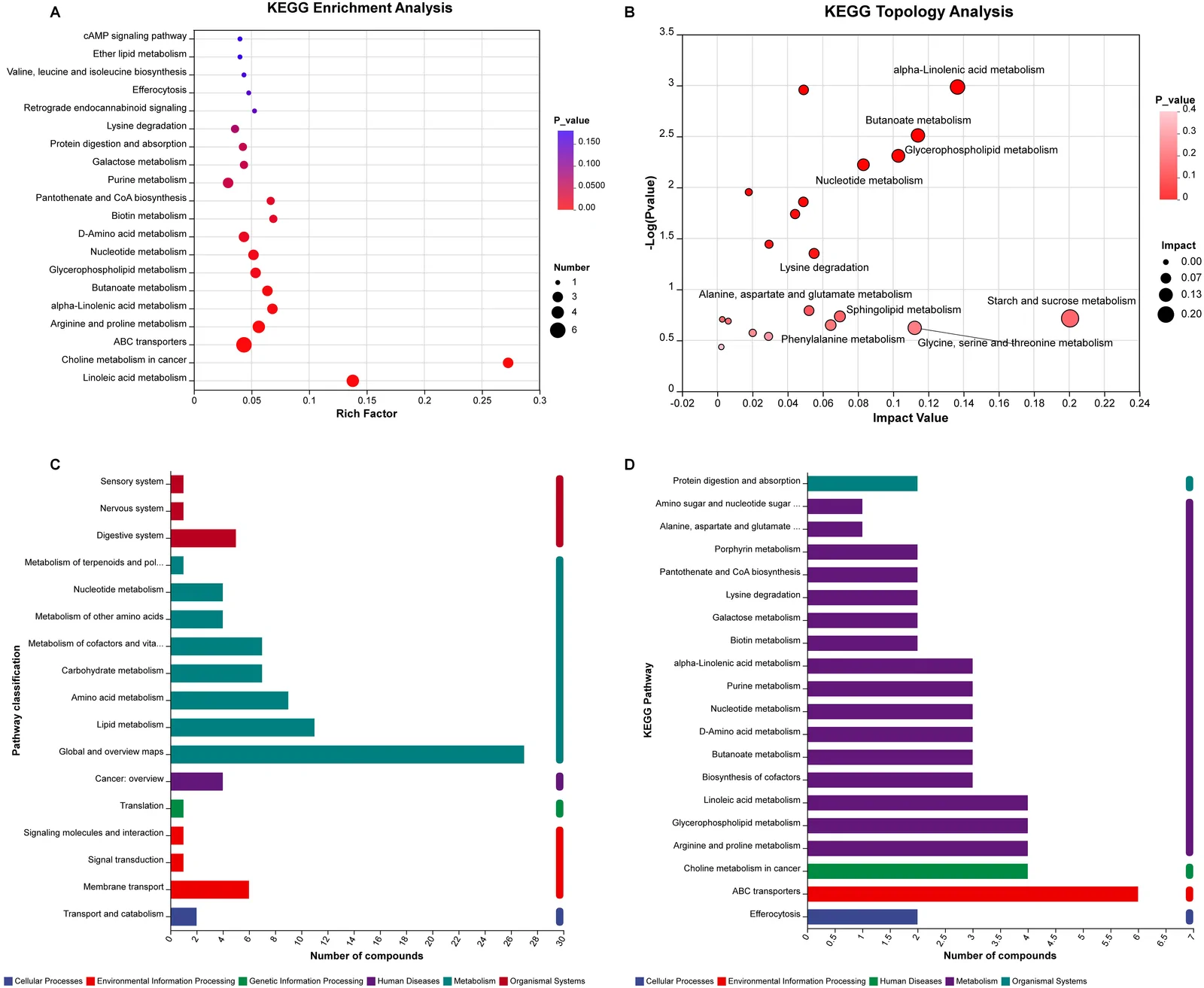
Metabolic pathway analysis and network visualization of serum differential metabolites between the HPIV and HCs groups. **(A)** Bubble plot of KEGG pathway enrichment analysis. **(B)** Bubble plot of KEGG topological analysis. **(C)** Classification of KEGG Level 2 pathways. **(D)** Classification of KEGG Level 3 pathways. Node color corresponds to the *P*-value, and node size indicates the pathway impact value.

At KEGG Level 2, the enriched pathways covered six major functional categories: metabolism, genetic information processing, environmental information processing, cellular processes, organismal systems, and human diseases (**Figure 5C**). At KEGG Level 3, the classification was more specific, encompassing 19 core pathways across five major categories, including carbohydrate, amino acid, lipid, and nucleotide metabolism, as well as pathways related to cellular function, protein digestion and absorption, and human diseases (**Figure 5D**).

### 3.7 Correlation between gut microbiota and serum metabolites

Procrustes analysis showed that the overall structural trends of the gut microbiota and serum metabolome were significantly correlated between the HPIV and healthy control groups (*P* = 0.001), and the combined dataset effectively distinguished the two groups (**Figure 6A**). These findings suggest that HPIV infection was associated with concurrent alterations in both gut microbiota composition and serum metabolite profiles, with the two omics layers exhibiting coordinated patterns of change. Two-way orthogonal partial least squares (O2PLS) analysis was performed to further explore the integrative associations between gut microbiota and serum metabolites (**Figure 6B**). The model showed a distinct separation between the HPIV and control groups along the first principal component (*P* < 0.05), supporting the presence of significant intergroup differences in the overall microbiota–metabolite association patterns.

**Figure 6:**
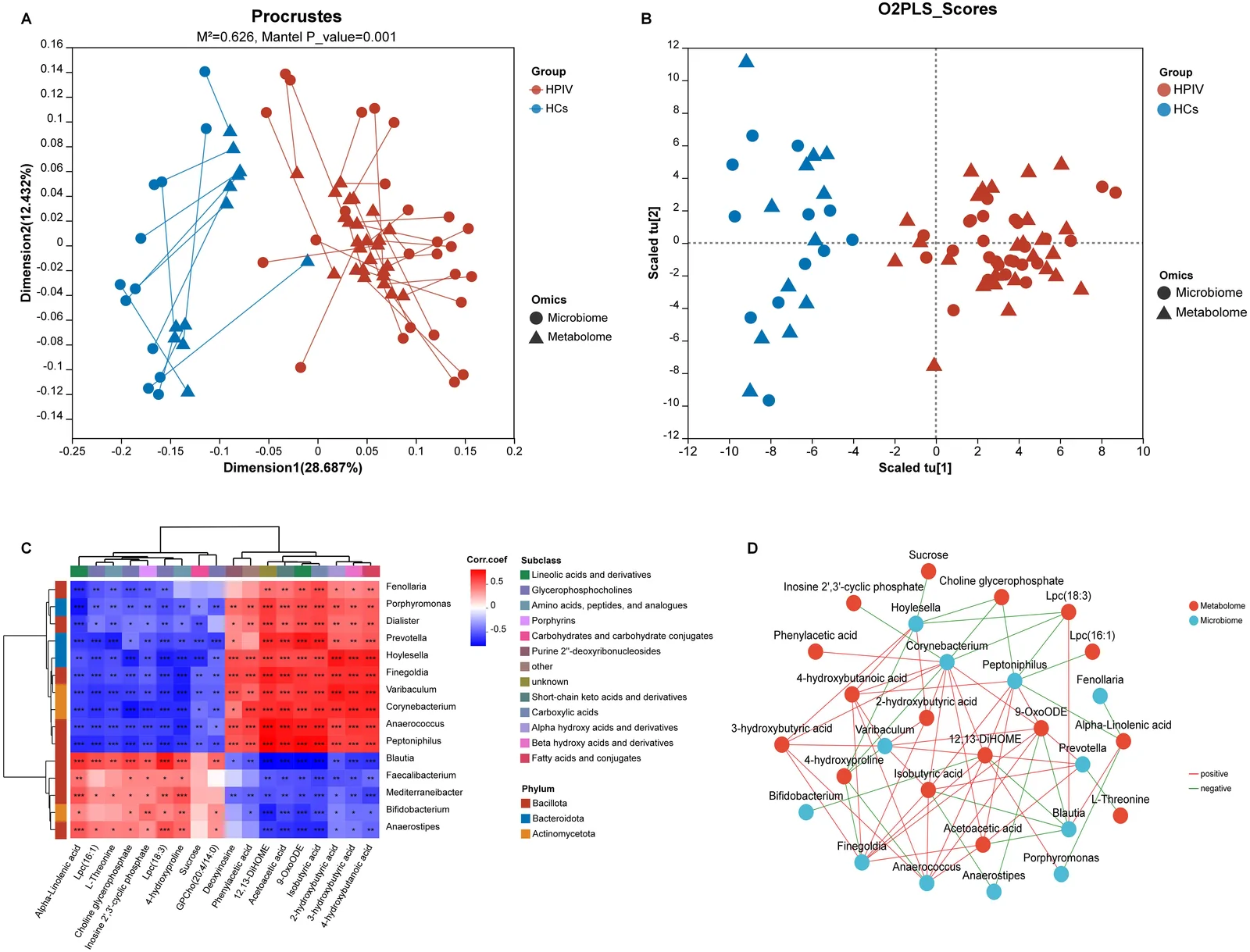
Correlation analysis between genus-level fecal microbial abundance and serum metabolite levels in the HPIV group and the HCs group. **(A, B)** Procrustes analysis and O2PLS score plots. **(C, D)** Spearman correlation heatmap and interaction network of microbiota–metabolite pairs. Correlation thresholds were set at |*r*| ≥ 0.6 and *P* < 0.05. Statistical significance was set at *P* < 0.05; *P < 0.05; **P < 0.01; ***P < 0.001.

Spearman correlation analysis was used to examine genus–metabolite associations, applying thresholds of |*r*| ≥ 0.6 and *P* < 0.05 for statistical significance (**Figure 6C**). The abundances of three opportunistic genera—*Anaerococcus*, *Peptoniphilus*, and *Finegoldia*—were positively correlated with up-regulated lipid metabolites (e.g., acetoacetic acid and 3-hydroxybutyric acid) and negatively associated with nutritional/protective metabolites (e.g., alpha-linolenic acid, sucrose, and L-threonine). Conversely, the abundance of *Blautia* showed the opposite correlation pattern, being negatively associated with the same lipid metabolites and positively associated with nutritional/protective metabolites. However, not all beneficial commensals exhibited similar correlation profiles; *Bifidobacterium* and *Faecalibacterium* did not show significant correlations with sucrose or L-threonine, suggesting potential functional heterogeneity among different beneficial taxa within this correlation network. Additionally, *Corynebacterium*, *Varibaculum*, and *Hoylesella* displayed broad-spectrum correlation patterns with multiple metabolites, potentially representing hub nodes in the microbiota–metabolite correlation network.

A microbiota–metabolite correlation network was constructed to visualize the pairwise associations between gut bacterial genera and serum metabolites (**Figure 6D**). The network exhibited core-enriched topological features, with certain genera correlating extensively with multiple differential metabolites and potentially serving as key nodes within the HPIV-associated correlation network.

### 3.8 Diagnostic performance of microbial and metabolic biomarkers

To evaluate the potential discriminatory capacity of the gut microbial and serum metabolic profiles identified in this study, we conducted receiver operating characteristic (ROC) curve analysis to assess their ability to distinguish children with HPIV infection from healthy controls.

Based on LEfSe analysis with LDA scores > 3.5, four core differentially abundant gut bacterial genera were identified as candidate microbial biomarkers: *Anaerococcus*, *Peptoniphilus*, *Finegoldia*, and *Blautia*. ROC analysis showed that the combined microbial biomarker panel yielded an AUC of 0.81 (95% CI: 0.68–0.94), suggesting moderate discriminatory performance for HPIV infection (**Figure 7A**). Additionally, five differential serum metabolites were screened by VIP values > 1 as candidate metabolic biomarkers, including 4-hydroxybutanoic acid, alpha-linolenic acid, choline glycerophosphate, L-threonine, and 4-hydroxyproline. The combined metabolite panel achieved an AUC of 0.98 (95% CI: 0.978–0.989), demonstrating relatively high discriminatory capacity for identifying HPIV infection in this exploratory cohort (**Figure 7B**).

**Figure 7:**
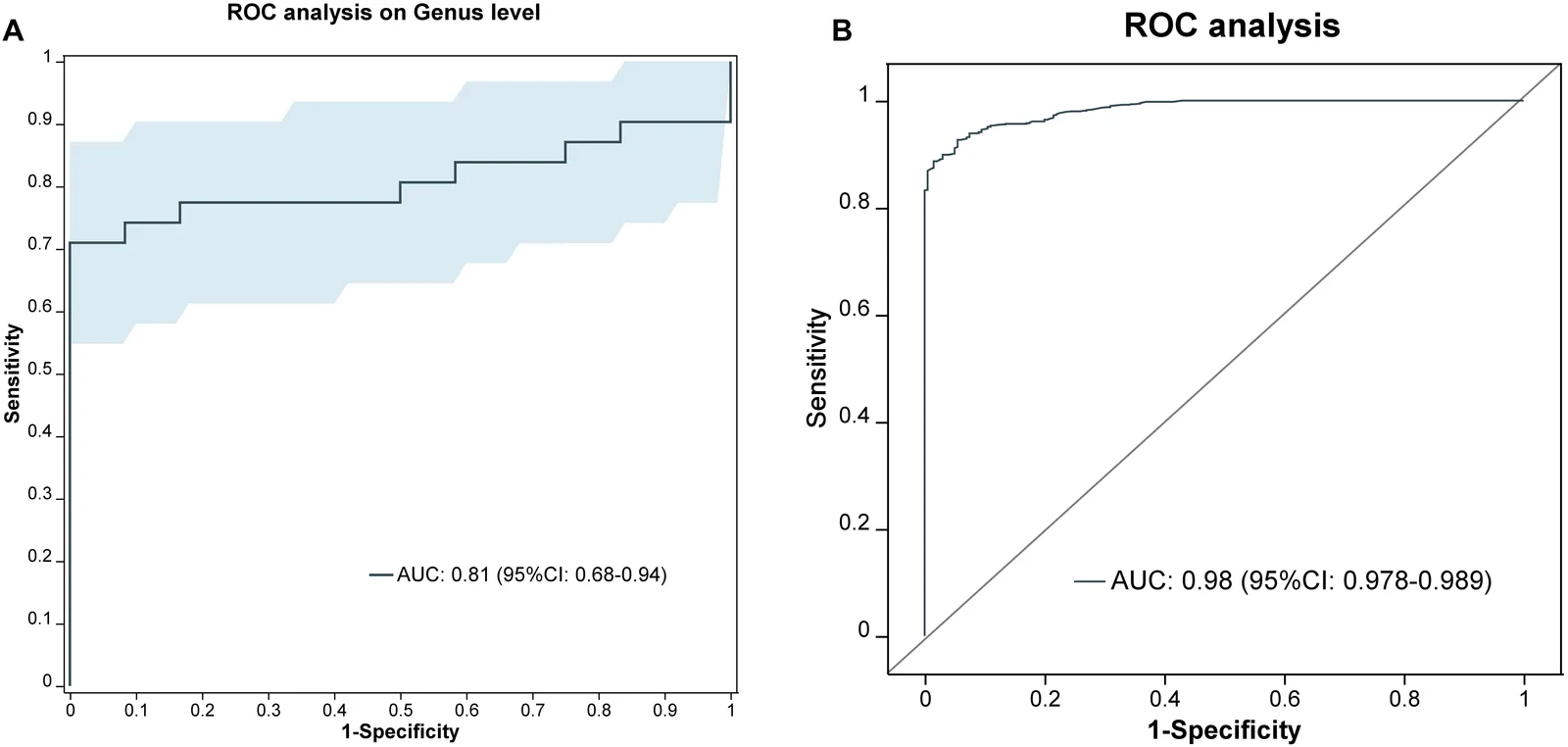
ROC curve analysis of candidate microbial and metabolic biomarkers for distinguishing HPIV infection. **(A)** ROC curve based on four differential gut bacterial genera. **(B)** ROC curve based on five differential serum metabolites. AUC values and corresponding 95% confidence intervals (CIs) are presented.

Notably, this ROC analysis is an exploratory evaluation performed in the same cohort used for biomarker screening. The reported AUC values may overestimate true diagnostic performance due to the lack of an independent external validation cohort, and the 95% confidence intervals are within-cohort estimates that do not reflect generalizability to external populations.

### 3.9 Exploratory analysis of hospitalization-related confounding

To evaluate whether hospitalization stress independently influenced the core multi-omic signatures, we performed an exploratory correlation analysis within the HPIV group (n = 31). Specifically, we assessed whether the abundance of the four key genera (*Anaerococcus*, *Peptoniphilus*, *Finegoldia*, and *Blautia*) and the levels of the five key serum metabolites (alpha-linolenic acid, choline glycerophosphate, L-threonine, 4-hydroxyproline, and 4-hydroxybutanoic acid) were associated with two proxy indicators of hospitalization-related physiological stress: length of hospital stay and pneumonia severity (mild vs. severe). Spearman correlation analysis revealed no significant associations for any of the tested variable–proxy pairs (all |*r*| ≤ 0.32, all *P* > 0.05; **Supplementary Table 9**). The largest absolute correlation was observed between *Anaerococcus* and length of stay (*r* = −0.32, *P* = 0.084), which approached but did not reach statistical significance. No consistent directional trends were observed across variables. These findings suggest that the observed gut dysbiosis and metabolic disturbances in HPIV children are unlikely to be predominantly driven by hospitalization duration or pneumonia severity per se within this cohort.

## 4 Discussion

In recent years, several studies have highlighted the complex regulatory mechanisms of the ‘gut–lung axis’ during viral respiratory infections. The intestinal and respiratory mucosae share characteristics of a common mucosal immune response; when infection occurs at a single mucosal site, it can affect immune homeostasis and microbiota balance in the intestinal mucosa through distal regulatory pathways (Dumas et al., 2018). Based on this gut–lung axis framework, the present study systematically investigated associations between HPIV respiratory infection and alterations in gut microbial diversity, microbiota composition, and serum metabolomic profiles in pediatric patients, providing preliminary evidence for exploring potential mechanisms underlying HPIV-associated gut–lung axis perturbations.

Among hospitalized children with HPIV infection, those with severe disease had longer hospital stays and were more likely to develop respiratory symptoms such as wheezing, tachypnea, and dyspnea. Furthermore, peripheral blood levels of WBC and PLT were significantly elevated in children with severe HPIV infection, whereas HGB levels were significantly lower, and these hematological changes were associated with gastrointestinal symptoms such as vomiting. Collectively, these clinical observations may provide preliminary clues for further investigations into the potential associations between gut microbiota alterations and systemic manifestations in children with HPIV infection.

Compared with healthy children, although the overall diversity of the gut microbiota in children with HPIV infection did not show significant changes, the composition of the microbiota exhibited distinct disease-specific alterations, with reduced microbiota health and structural homeostasis, consistent with significant gut microbiota dysbiosis—findings that align with previous observations in COVID-19 and H1N1 virus infections (Gu et al., 2020; Liao et al., 2024). In children with HPIV infection, the relative abundance of opportunistic pathogens in the gut increased significantly, including genera such as *Anaerococcus*, *Peptoniphilus*, and *Finegoldia*, while the relative abundance of beneficial human commensal bacteria—such as *Bifidobacterium*, *Blautia*, and *Faecalibacterium*—decreased significantly. Gut microbiota dysbiosis is often associated with inflammatory and autoimmune diseases. Studies have reported that anaerobic bacteria (e.g., *Peptoniphilus*, *Finegoldia*, and *Anaerococcus*) are primary colonizers of wound infections and can trigger endogenous infections when the host’s immune function is compromised (Murphy and Frick, 2013). In the intestines of patients with constipation, the abundance of *Blautia* is reduced, and *Bifidobacterium* has been shown to eliminate intestinal pathogens and reduce the body’s inflammatory response (Reading and Kasper, 2011). *Faecalibacterium* is considered an anti-inflammatory commensal capable of counteracting various gastrointestinal diseases (Leylabadlo et al., 2020).

There are significant differences in serum metabolic profiles between children with HPIV infection and healthy children; HPIV infection was associated with marked serum metabolic disturbances. Specifically, children with HPIV infection exhibited 579 significantly upregulated metabolites and 211 significantly downregulated metabolites. Levels of acetoacetic acid, 3-hydroxybutyric acid, isobutyric acid, and 4-hydroxybutanoic acid were significantly elevated in the serum of children with HPIV infection. Results from KEGG pathway enrichment analysis showed that these metabolites were significantly enriched in the butanoate metabolism pathway, which primarily involves the biosynthesis and degradation of SCFAs and plays a role in maintaining intestinal barrier function, as well as anti-inflammatory and immunomodulatory effects. Acetoacetic acid, as one of the three major ketone bodies, provides energy to peripheral tissues such as the brain and heart; 3-hydroxybutyric acid, as an important signaling molecule, inhibits oxidative stress and exerts epigenetic regulatory functions; together, they form the core of ketone body metabolism, are chemically interconvertible, and act synergistically in physiological functions (Najac et al., 2019). Isobutyric acid primarily originates from the catabolism of the branched-chain amino acid valine; 4-hydroxybutanoic acid, as a neurotransmitter in the central nervous system, is metabolized primarily in association with γ-aminobutyric acid (GABA) and also participates in various specialized carbon fixation cycles in microorganisms (Wei et al., 2023).

The levels of alpha-linolenic acid, choline glycerophosphate, sucrose, L-threonine, and 4-hydroxyproline were significantly downregulated in the serum of children with HPIV infection. KEGG pathway enrichment analysis revealed that alpha-linolenic acid, a core substrate of the alpha-linolenic acid metabolism pathway, has been reported to alleviate pulmonary inflammatory damage by inhibiting key inflammatory pathways such as NF-κB (Pisoschi et al., 2022); its downregulation may reflect depletion of substrates for the synthesis of anti-inflammatory lipid mediators, which may be associated with HPIV-associated immune evasion and the formation of a pro-inflammatory environment (Kumar et al., 2016; Rojas et al., 2021). Choline glycerophosphate was enriched in the glycerophospholipid metabolism pathway. As a core product of phospholipid hydrolysis, its downregulation may reflect HPIV-associated remodeling of host cell membrane phospholipid metabolism to meet the demands of viral envelope synthesis and replication, while simultaneously affecting host cell signaling (Yuan et al., 2022). Sucrose was significantly enriched in the starch and sucrose metabolism pathway, which primarily regulates carbohydrate metabolism, glycolysis, and gluconeogenesis (Borewicz, 2018). The downregulation of sucrose metabolism may be associated with energy metabolism reprogramming and changes in the carbohydrate composition of the airway surface fluid layer in the context of HPIV infection, which may affect the host’s mucosal defense function (Fan et al., 2022). L-threonine and 4-hydroxyproline were significantly enriched in the glycine, serine, and threonine metabolism pathway. Their concurrent downregulation suggests that HPIV infection may be accompanied by disruptions to host protein synthesis, energy metabolism, and extracellular matrix homeostasis, which may be closely associated with airway tissue injury and defective tissue repair after viral infection (Al-Shalan et al., 2023).

Based on the results of association analyses between gut bacterial genera and serum metabolites, alterations in gut microbiota and serum metabolites were correlated with remote immune and inflammatory profiles in children with HPIV infection through complex immune-metabolic networks, consistent with the patterns proposed in previous studies (Zhu et al., 2025). Elevated abundances of genera such as *Anaerococcus* were correlated with reduced alpha-linolenic acid levels, which have been previously associated with pro-inflammatory processes and impaired tissue repair (Cianciosi et al., 2024). Higher *Blautia* abundance was associated with preserved alpha-linolenic acid levels; this correlation may be related to its reported role in maintaining intestinal barrier integrity, as suggested in previous studies (Kim et al., 2020). Elevated abundances of *Anaerococcus*, *Peptoniphilus*, and *Finegoldia* were correlated with markers suggestive of impaired intestinal epithelial and mucus barriers in pediatric patients. Reductions in L-threonine and 4-hydroxyproline may be indicative of increased consumption for mucin and collagen synthesis during tissue repair (Camps et al., 2025). Reduced choline glycerophosphate levels were also correlated with potential bacterial choline utilization by genera such as *Anaerococcus* as a possible mechanism to evade host immune recognition and enhance adhesion, thereby depleting the host’s choline pool, as hypothesized in prior research.

*Blautia* is known to produce short-chain fatty acids (SCFAs) via dietary fiber fermentation, a well-established mechanism for maintaining gut microbiota homeostasis. Among these SCFAs, acetate has been shown to enhance antiviral innate immunity through activation of the type I interferon pathway, while butyrate promotes regulatory T cell (Treg) differentiation via histone deacetylase (HDAC) inhibition, thereby limiting excessive inflammatory responses (Kumar et al., 2021). These SCFA-mediated pathways provide a potential mechanistic link between gut microbial metabolites and pulmonary immune regulation, consistent with the gut–lung axis concept, although direct evidence in the HPIV context remains to be established.

This study ultimately screened and identified four differentially abundant gut bacterial genera (*Anaerococcus*, *Peptoniphilus*, *Finegoldia*, and *Blautia*) and five serum metabolites (alpha-linolenic acid, choline glycerophosphate, L-threonine, 4-hydroxyproline, and 4-hydroxybutanoic acid) as candidate biomarker panels. We constructed a combined microbial biomarker model and a combined metabolite biomarker model to distinguish hospitalized children with HPIV infection from healthy children. In this exploratory cohort, the AUC values for the two models were 0.81 and 0.98, respectively. The microbial composite biomarker showed exploratory discriminatory performance, while the metabolite composite biomarker exhibited relatively high distinguishing capacity within this cohort. These findings provide preliminary exploratory evidence for the development of candidate biomarkers for HPIV infection; however, given the exploratory nature of this analysis and the lack of independent validation, these results should be interpreted with caution and warrant validation in larger, independent cohorts.

This study still has certain limitations.

First, this study was a single-center retrospective investigation with a relatively limited sample size, and the sample size was imbalanced between the HPIV and control groups (31 cases vs. 12 controls). Although all control subjects were recruited from the same hospital and during the same period as the HPIV group, and were screened using identical eligibility criteria, the fact that these samples were also used in parallel studies by our research team may nevertheless introduce residual selection bias. Post-hoc power analysis indicated that the power to detect a moderate effect (*d* = 0.5) was only 0.30; therefore, negative findings—such as those for α-diversity indices—should be interpreted with caution, as they may reflect limited statistical sensitivity rather than the true absence of difference. In contrast, the core positive findings all exhibited large effect sizes with adequate power: *Anaerococcus* (*d* = 1.40, power = 0.98), *Peptoniphilus* (*d* = 1.69, power > 0.99), *Finegoldia* (*d* = 1.64, power > 0.99), and *Blautia* (*d* = 4.90, power > 0.99). These findings remained robust across permutation tests and cross-validation.

Second, clinical metadata were incomplete. Key clinical indicators, including HPIV subtype, viral load, and blood oxygen saturation, were not incorporated into the analysis, which limits our ability to explore the associations between gut microbiota and metabolites and disease severity. Furthermore, nasopharyngeal PCR testing was not performed in the control group; although active infection was excluded based on clinical manifestations and routine blood tests, the possibility of asymptomatic subclinical infection cannot be fully ruled out. Finally, some patients in the HPIV group presented with gastrointestinal symptoms, and the independent effect of these symptoms on the gut microbiota cannot be fully excluded.

Third, confounding factors were not fully controlled in this study. Although we excluded participants with antibiotic/probiotic use and chronic diseases via strict eligibility criteria to control for core confounders, we did not collect data on breastfeeding history, delivery mode, dietary habits, or use of non-antibiotic medications. Importantly, we lacked a disease control group comprising children hospitalized with non-HPIV respiratory infections; therefore, we cannot fully discriminate HPIV-specific microbiome and metabolome shifts from changes common to acute respiratory infection or hospitalization stress. Nevertheless, our additional exploratory analysis partially mitigates concerns regarding hospitalization-related confounding.

Fourth, there are intrinsic methodological limitations. LEfSe analysis is susceptible to compositional bias and data sparsity; thus, the differentially abundant taxa identified by this approach should be regarded as exploratory findings that require further validation via complementary methods such as qPCR or metagenomic sequencing. Furthermore, metabolite annotation was performed at MSI Level 2 (putative annotation via MS/MS spectral matching) rather than confirmed with authentic standards, which reduces the confidence of metabolite identification. In addition, the diagnostic performance of the identified biomarkers was evaluated in the same cohort used for feature selection, with no nested cross-validation or independent validation cohort. The reported AUC values, particularly the metabolite panel AUC of 0.98, may be overoptimistic due to overfitting bias. Larger prospective studies with independent validation are needed to verify their generalizability, diagnostic value, and performance relative to routine clinical indicators.

Fifth, this study is an observational associative study based on multi-omics profiling. Correlation analyses including Procrustes, O2PLS, and Spearman correlation cannot establish causal relationships among gut microbiota dysbiosis, serum metabolic alterations, and HPIV pathogenesis. The proposed regulatory mechanisms based on the gut–lung axis remain speculative and require further validation through *in vivo* and *in vitro* functional experiments. In future prospective studies, we will collect sufficient independent samples and perform targeted LC-MS/MS assays to validate the key metabolite biomarkers identified in this study and further explore their biological functions during HPIV infection.

## 5 Conclusion

In summary, this study systematically investigated gut microbiota and serum metabolomic profiles in children with HPIV infection and their associations with gut–lung axis pathways. HPIV infection was associated with respiratory symptoms and intestinal alterations, accompanied by gut microbiota shifts characterized by enrichment of opportunistic pathogens (*Anaerococcus*, *Peptoniphilus*, *Finegoldia*) and depletion of beneficial commensals (*Bifidobacterium*, *Blautia*, *Faecalibacterium*). Serum metabolomic profiling revealed marked disturbances, with 579 upregulated and 211 downregulated metabolites, primarily enriched in pathways related to alpha-linolenic acid, butanoate, glycerophospholipid, starch/sucrose, and glycine/serine/threonine metabolism. Integration of multi-omics data showed that opportunistic pathogens positively correlated with pro-inflammatory lipid metabolites (e.g., acetoacetic acid, 3-hydroxybutyric acid) and negatively correlated with protective metabolites (e.g., alpha-linolenic acid, sucrose, L-threonine), while *Blautia* exhibited the opposite pattern. Four key genera and five metabolites demonstrated exploratory discriminatory capacity for HPIV infection (AUC: 0.81 and 0.98, respectively) within this cohort, serving as candidate biomarkers for further validation. These findings suggest that gut microbiota and metabolic pathways may represent exploratory research directions for future intervention studies; however, the potential clinical translational value of these targeted strategies remains hypothetical. Given the exploratory and hypothesis-generating nature of this study, further validation is warranted through larger sample sizes, independent cohorts, prospective longitudinal studies, and mechanistic experiments to confirm the observed associations and evaluate their clinical applicability.

## Data Availability

The data presented in this study are deposited in the NCBI BioProject repository, accession number PRJNA1496541. The data can be accessed at: https://www.ncbi.nlm.nih.gov/bioproject/PRJNA1496541.
