# Supplementary material for: Gut microbiota and serum metabolomics in children with human parainfluenza virus infection
Source: Front Cell Infect Microbiol. 2026 Oct 5;16:1941674. doi: 10.3389/fcimb.2026.1941674 (PMC13677554; doi:10.3389/fcimb.2026.1941674)
Supplement: Supplementary file 1 [file DataSheet1.pdf]

Supplementary Material

1 Supplementary Figures and Tables

1.1 Supplementary Figures

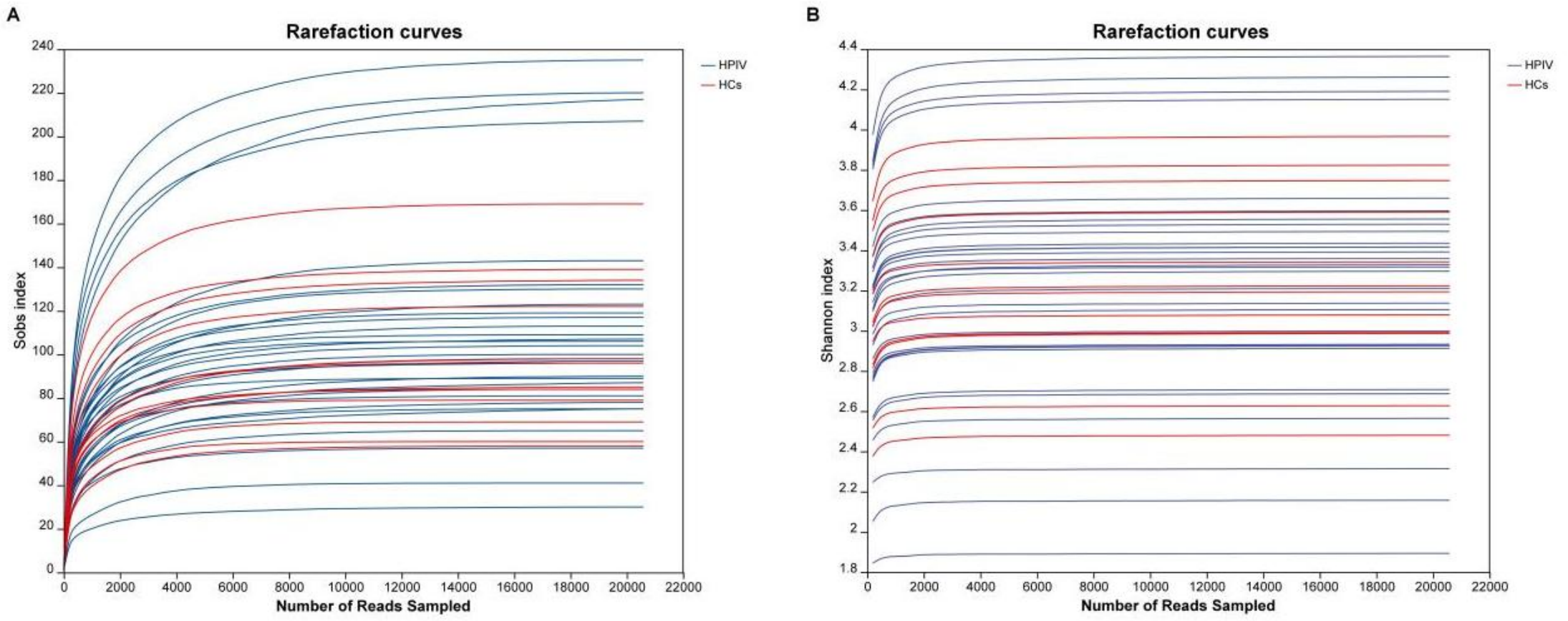

Supplementary Figure 1. Rarefaction curve analysis of gut microbiota in the HPIV group and the HCs group. (A) Sobs index curves reflecting bacterial community richness; (B) Shannon index curves showing bacterial community diversity.

1.2 Supplementary Tables

Supplementary Table 1. Software and versions used for 16S rRNA gene sequencing data analysis.

Analysis Software	Version	Database Link
fastp	0.23.4	https://github.com/OpenGene/fastp
FLASH	1.2.11	https://ccb.jhu.edu/software/FLASH/index.shtml
Qiime2	2024	https://qiime2.org
DADA2	2024	https://qiime2.org
Deblur	2024	https://benjjneb.github.io/dada2/
Mega	7	https://www.megasoftware.net/
SILVA	138	https://www.arb-silva.de/
UNITE	8	https://unite.ut.ee/
RDP	11.5	http://rdp.cme.msu.edu/
GreenGenes	135	http://greengenes.secondgenome.com/
FunGene	9.6	http://www.fungene-db.fr/
MaarjAM	81	https://www.maarjam.botany.ut.ee/
HPB	none	https://www.cerl.org/resources/hpb/content
PR2	1	https://github.com/vaulot/pr2_database
Funguild	1	http://www.funguild.org/

Tax4fun	0.3.1	http://tax4fun.gobics.de/
MAFFT	7.2	https://mafft.cbrc.jp/alignment/software/
IQ-TREE	1.6.8	http://www.iqtree.org/
PICRUSt2	2.2.0	https://github.com/picrust/picrust2/
Mothur	1.48.3	–

Supplementary Table 2. Software versions for metabolomic data analysis.

Analysis Item	Software	Software Version	Software Parameters
Expression data preprocessing	Majorbio self-developed software	–	None
PCA analysis	ropls (R package)	1.6.2	Default parameters
Differential metabolite analysis (multivariate statistics)	ropls (R package)	1.6.2	Default parameters
KEGG compound classification	KEGG	kegg_v20241007; original Release 2017-05-01	None
KEGG functional pathways	KEGG	kegg_v20241007; original Release 2017-05-01	None
Venn diagram	VennDiagram (R package)	1.6.20	None
KEGG pathway enrichment analysis	scipy (Python)	1.0.0	*P*-value < 0.05
Heatmap	scipy (Python)	1.0.0	None
Cluster analysis	scipy (Python)	1.0.0	None
Correlation analysis	scipy (Python)	1.0.0	None
VIP analysis	ropls (R); scipy (Python)	1.6.2; 1.0.0	None
iPath metabolic pathway analysis	iPath 3.0	3.0	None
ROC curve analysis	pROC (R package)	1.12.1	None

Supplementary Table 3. Univariate and multivariate logistic regression analyses of factors associated with severe HPIV infection.

Variable	Univariate analysis		Multivariate adjusted analysis	
	OR (95% CI)	*P*-value	OR (95% CI)	*P*-value
Sex (male vs. female)	0.87 (0.48, 1.57)	0.645	1.20 (0.59, 2.46)	0.614
Residence (urban vs. rural)	0.67 (0.37, 1.22)	0.192	0.96 (0.46, 1.98)	0.906
Age (reference: <1 year)	1.00	—	1.00	—
1 to < 3 years	0.59 (0.29, 1.19)	0.144	1.64 (0.65, 4.09)	0.292
3 to < 5 years	0.33 (0.15, 0.72)	0.006	1.21 (0.44, 3.32)	0.717
≥ 5 years	0.16 (0.04, 0.58)	0.005	0.39 (0.08, 1.80)	0.227
Vomiting (yes vs. no)	2.36 (1.15, 4.84)	0.019	2.58 (1.08, 6.14)	0.033
Wheezing (yes vs. no)	3.24 (1.55, 6.79)	0.002	—	—
Tachypnea (yes vs. no)	13.83 (4.92, 38.86)	<0.001	—	—
Dyspnea (yes vs. no)	13.67 (6.57, 28.43)	<0.001	14.56 (6.19, 34.23)	<0.001

HGB level (per 1 g/L increase)	0.97 (0.95, 0.99)	0.009	0.98 (0.95, 1.00)	0.045
PLT count (per 10^9/L increase)	1.00 (1.00, 1.01)	0.004	—	—
WBC count (per 10^9/L increase)	1.07 (1.01, 1.14)	0.020	—	—

Note: The multivariate logistic regression model was adjusted for sex, age, and residence. "—" indicates the variable was not retained in the final multivariate model after stepwise regression (entry $P < 0.15$, removal $P > 0.151$). Multicollinearity was assessed using variance inflation factors (VIF), and no significant collinearity was detected (all VIF values ≤ 1.77, mean VIF = 1.28). Model calibration was assessed using the Hosmer–Lemeshow test ($\chi^2 = 13.32$, $P = 0.101$), and model discrimination was evaluated using the area under the receiver operating characteristic curve (AUC = 0.815, 95% CI: 0.745–0.884).

Supplementary Table 4. Comparison of clinical characteristics between included (n = 31) and non–included (n = 199) HPIV hospitalized patients.

Variable	Included (n = 31)	Non–included (n = 199)	*P*-value
Sex, n (%)			0.234
Male	16 (51.61)	125 (62.81)	
Female	15 (48.39)	74 (37.19)	
Place of residence, n (%)			0.180
Urban	22 (70.97)	116 (58.29)	
Rural	9 (29.03)	83 (41.71)	
Age (years), n (%)			0.716
<1 year	6 (19.35)	54 (27.14)	
1 to < 3 years	11 (35.48)	66 (33.17)	
3 to < 5 years	11 (35.48)	54 (27.14)	
≥ 5 years	3 (9.68)	25 (12.56)	
Length of hospitalization (days), median (IQR)	4 (3, 6)	5 (4, 6)	0.054
Cough duration (days), median (IQR)	6 (2, 10)	5 (2, 10)	0.402
Fever duration (days), median (IQR)	3 (1, 4)	3 (1, 5)	0.920
Clinical symptoms, n (%)			
Sputum production	25 (80.65)	135 (67.84)	0.149
Nasal congestion	21 (67.74)	137 (68.84)	0.902
Rhinorrhea	22 (70.97)	134 (67.34)	0.687
Convulsion	2 (6.45)	11 (5.53)	0.689
Wheezing	7 (22.58)	28 (14.07)	0.279
Tachypnea	3 (9.68)	22 (11.06)	0.819
Dyspnea	5 (16.13)	46 (23.12)	0.384
Severe pneumonia	7 (22.58)	59 (39.65)	0.418
Laboratory indicators, median (IQR)			
HGB (g/L)	119 (113, 127)	117 (110, 124)	0.495
PLT (10^9/L)	309 (211, 357)	292 (228, 393)	0.551
WBC (10^9/L)	8.23 (6.01, 12.26)	9.17 (6.47, 12.37)	0.824
NEUT (10^9/L)	3.60 (2.60, 4.86)	3.85 (2.31, 6.17)	0.849

LYMPH (10^9/L)	3.78 (2.79, 4.99)	3.78 (2.11, 5.32)	0.860
CRP (mg/L)	4.53 (1.93, 10.93)	5.36 (0.97, 13.09)	0.849
PCT (ng/mL)	0.13 (0.10, 0.22)	0.13 (0.07, 0.27)	0.499
IL-6 (pg/mL)	14.76 (7.58, 22.87)	12.27 (4.83, 29.00)	0.306

Supplementary Table 5. Summary of effective read counts and number of ASVs for each sample.

Sample ID	Number of ASVs	Effective reads	Sample ID	Number of ASVs	Effective reads
A01	106	26102	A23	114	34635
A02	117	31034	A25	57	31755
A03	100	30943	A26	81	32222
A04	107	30196	A27	104	32999
A05	75	26288	A28	75	47504
A06	123	42866	A29	87	37429
A07	41	28062	A30	98	34069
A08	109	31685	A31	96	30814
A09	208	40965	A32	97	39808
A10	220	43383	B01	69	34640
A11	84	36011	B02	98	31683
A12	78	28706	B03	135	28414
A13	218	47496	B04	96	30109
A14	132	34517	B05	84	28645
A15	119	36071	B06	58	26811
A16	30	27644	B07	169	33480
A17	131	35019	B08	139	29841
A18	65	32462	B09	85	32283
A19	143	33000	B10	122	29480
A20	90	33670	B11	60	20590
A21	89	33735	B12	79	26182
A22	235	36340			

Note: The number of ASVs denotes amplicon sequence variants retrieved following sequence denoising.

Supplementary Table 6. PERMANOVA results of gut microbial community composition.

Factor	Df	Sum Sq	Mean Sq	F	R^2	P-value
Group (HPIV vs. HCs)	1	3.54	3.54	17.77	0.296	0.001
Sex (male vs. female)	1	0.15	0.15	0.73	0.012	0.614
Age (years)	3	0.90	0.30	1.50	0.075	0.099
Residuals	37	7.37	—	—	0.617	—
Total	42	11.96	—	—	1.000	—

Notes: PERMANOVA was performed using Bray–Curtis distances with 999 permutations. R^2 represents the proportion of variance explained by each factor. '—' indicates not applicable. Age was categorized into four groups: <1 year, 1 to <3 years, 3 to <5 years, and ≥5 years (Df = 3).

Supplementary Table 7. List of 37 differential metabolites with VIP > 1 in children with HPIV infection.

Metabolite	VIP	FC	Log_2FC	*P*-value	FDR *q*-value	Regulation
Galactosylglycerol	2.505	1.973	0.980	9.13E-09	2.39E-07	Up
Vernolic acid	2.454	1.344	0.427	1.05E-12	1.48E-10	Up
Biocytin	2.425	1.720	0.782	2.97E-08	6.08E-07	Up
Isobutyric acid	2.338	1.432	0.518	4.94E-11	3.35E-09	Up
9-OxoODE	2.177	1.263	0.337	1.49E-11	1.30E-09	Up
4-hydroxybutanoic acid	2.109	1.602	0.680	1.57E-06	1.49E-05	Up
5'-Phosphoribosyl-N-formylglycinamidine	2.098	1.405	0.490	1.36E-07	2.04E-06	Up
4'-Phosphopantothenoylcysteine	2.094	0.722	-0.469	4.30E-04	1.47E-03	Down
Alpha-Linolenic acid	1.913	0.799	-0.325	8.45E-05	3.70E-04	Down
12,13-DiHOME	1.753	1.132	0.179	1.87E-11	1.56E-09	Up
3-hydroxybutyric acid	1.739	1.138	0.186	9.12E-08	1.48E-06	Up
N-acetylaspartylglutamic acid	1.711	1.239	0.309	9.45E-08	1.52E-06	Up
Deoxyguanosine	1.672	1.297	0.375	5.69E-06	4.16E-05	Up
Farnesylcysteine	1.643	1.553	0.635	7.70E-03	1.86E-02	Up
Galactocerebroside	1.568	1.131	0.177	2.66E-09	8.71E-08	Up
Erythritol	1.542	1.124	0.168	9.02E-08	1.46E-06	Up
Inosine 2',3'-cyclic phosphate	1.477	0.814	-0.297	2.20E-04	8.20E-04	Down
Phenylacetic acid	1.426	1.562	0.643	6.30E-04	2.07E-03	Up
Pimelic acid	1.411	1.309	0.388	7.40E-04	2.37E-03	Up
Nicotinic acid ribonucleoside	1.393	0.756	-0.404	1.20E-04	4.90E-04	Down
Chitin	1.367	1.112	0.153	5.36E-09	1.57E-07	Up
Gamma-glutamyl-L-putrescine	1.346	0.876	-0.192	2.10E-04	7.80E-04	Down
DCMP	1.339	0.885	-0.177	2.89E-05	1.60E-04	Down
Acetoacetic acid	1.288	1.130	0.177	4.92E-11	3.34E-09	Up
Spermine	1.272	1.247	0.319	2.23E-03	6.30E-03	Up
Lpc(16:1)	1.252	0.935	-0.097	5.49E-09	1.60E-07	Down
L-Threonine	1.235	0.912	-0.133	2.674E-8	5.593E-7	Down
9-Oxo-nonanoic acid	1.229	1.113	0.155	6.89E-06	4.85E-05	Up
Deoxyinosine	1.227	1.202	0.266	1.35E-03	4.02E-03	Up
GPCho(20:4/14:0)	1.222	0.899	-0.153	2.10E-04	7.80E-04	Down
4-hydroxyproline	1.198	0.908	-0.140	3.28E-05	1.70E-04	Down
Sucrose	1.172	0.918	-0.124	9.53E-05	4.10E-04	Down
Lpc(18:3)	1.162	0.937	-0.094	1.83E-06	1.67E-05	Down
5-Aminovaleric Acid	1.127	1.117	0.159	1.76E-03	5.09E-03	Up
3-(3-indolyl)-2-oxopropanoic acid	1.098	0.888	-0.171	5.97E-07	6.79E-06	Down
Choline glycerophosphate	1.083	0.944	-0.083	3.62E-06	2.89E-05	Down

2-hydroxybutyric acid	1.000	1.048	0.068	3.63E-06	2.89E-05	Up

Note: VIP, variable importance in projection from the OPLS-DA model; FC, fold change (HPIV/HCs); FDR *q*-value, false discovery rate adjusted using the Benjamini–Hochberg method. Metabolites with VIP > 1 and FDR *q*-value < 0.05 were considered significantly differential. Upregulation or downregulation is relative to the healthy control group. Abbreviations: DCMP, 2′-deoxycytidine monophosphate; GPCho, glycerophosphocholine; Lpc, lysophosphatidylcholine; ODE, octadecadienoic acid; DiHOME, dihydroxyoctadecenoic acid.

Supplementary Table 8. Diagnostic performance of 37 key serum metabolites for pediatric HPIV infection.

Metabolite	AUC	95% CI
Galactosylglycerol	1.00	(0.75, 1.00)
Biocytin	1.00	(0.85, 1.00)
4-hydroxybutanoic acid	1.00	(0.86, 1.00)
Phenylacetic acid	0.97	(0.60, 1.00)
Farnesylcysteine	0.75	(0.27, 1.00)
Isobutyric acid	1.00	(0.85, 1.00)
5'-Phosphoribosyl-N-formylglycinamidine	1.00	(0.76, 1.00)
Vernolic acid	1.00	(0.89, 1.00)
Pimelic acid	0.69	(0.22, 1.00)
Deoxyguanosine	1.00	(0.78, 1.00)
9-OxoODE	1.00	(0.85, 1.00)
Spermine	0.75	(0.42, 1.00)
N-acetylaspartylglutamic acid	1.00	(0.80, 1.00)
Deoxyinosine	0.97	(0.52, 1.00)
3-hydroxybutyric acid	1.00	(0.80, 1.00)
12,13-DiHOME	1.00	(0.85, 1.00)
Galactocerebroside	1.00	(0.78, 1.00)
Acetoacetic acid	1.00	(0.91, 1.00)
Erythritol	1.00	(0.90, 1.00)
5-Aminovaleric Acid	0.66	(0.45, 1.00)
9-Oxo-nonanoic acid	0.94	(0.70, 1.00)
Chitin	1.00	(0.81, 1.00)
2-hydroxybutyric acid	1.00	(0.74, 1.00)
4'-Phosphopantothenoylcysteine	1.00	(0.64, 1.00)
Nicotinic acid ribonucleoside	0.81	(0.55, 1.00)
Alpha-Linolenic acid	0.94	(0.55, 1.00)
Inosine 2',3'-cyclic phosphate	0.94	(0.63, 1.00)
Gamma-glutamyl-L-putrescine	1.00	(0.50, 1.00)
DCMP	1.00	(0.74, 1.00)

3-(3-indolyl)-2-oxopropanoic acid	0.97	(0.78, 1.00)
GPCho(20:4/14:0)	0.69	(0.55, 1.00)
4-hydroxyproline	0.82	(0.70, 1.00)
L-Threonine	1.00	(0.78, 1.00)
Sucrose	0.69	(0.59, 1.00)
Lpc(16:1)	1.00	(0.86, 1.00)
Lpc(18:3)	0.91	(0.74, 1.00)
Choline glycerophosphate	1.00	(0.68, 1.00)

Supplementary Table 9. Spearman correlation coefficients (*r*) and *P*-values between hospitalization-related factors and the four core bacterial genera and five key serum metabolites in the HPIV group (n = 31).

Variable	Length of hospitalization		Pneumonia severity	
	r (95% CI)	*P*-value	*r* (95% CI)	*P*-value
Four key gut bacterial genera				
Anaerococcus	-0.32 (-0.69, 0.04)	0.084	-0.25 (-0.65, 0.14)	0.207
Finegoldia	-0.28 (-0.66, 0.10)	0.146	-0.13 (-0.53, 0.27)	0.528
Peptoniphilus	-0.18 (-0.57, 0.21)	0.366	-0.21 (-0.53, 0.12)	0.212
Blautia	0.12 (-0.29, 0.52)	0.569	-0.02 (-0.41, 0.38)	0.932
Five key serum metabolites				
Alpha-linolenic acid	0.03 (-0.37, 0.42)	0.902	-0.07 (-0.41, 0.28)	0.701
Choline glycerophosphate	-0.04 (-0.46, 0.37)	0.839	0.18 (-0.14, 0.51)	0.272
L-threonine	0.06 (-0.35, 0.46)	0.785	0.07 (-0.32, 0.45)	0.732
4-hydroxyproline	-0.05 (-0.48, 0.37)	0.813	-0.04 (-0.42, 0.33)	0.824
4-hydroxybutanoic acid	0.13 (-0.22, 0.49)	0.463	0.10 (-0.27, 0.48)	0.587

Notes: Values in parentheses are 95% confidence intervals derived from bootstrap resampling (1,000 replications).
